# The burden of schizophrenia in the Middle East and North Africa region, 1990–2019

**DOI:** 10.1038/s41598-024-59905-8

**Published:** 2024-04-27

**Authors:** Saeid Safiri, Maryam Noori, Seyed Aria Nejadghaderi, Ali Shamekh, Mark J. M. Sullman, Gary S. Collins, Ali-Asghar Kolahi

**Affiliations:** 1 Neurosciences Research Center, Aging Research Institute, Tabriz University of Medical Sciences, Tabriz, Iran; 2 Clinical Research Development Unit of Tabriz Valiasr Hospital, Tabriz University of Medical Sciences, Tabriz, Iran; 3 Student Research Committee, School of Medicine, Iran University of Medical Sciences, Tehran, Iran; 4 HIV/STI Surveillance Research Center, WHO Collaborating Center for HIV Surveillance, Institute for Futures Studies in Health, Kerman University of Medical Sciences, Kerman, Iran; 5 Systematic Review and Meta-analysis Expert Group (SRMEG), Universal Scientific Education and Research Network (USERN), Tehran, Iran; 6 Student Research Committee, Tabriz University of Medical Sciences, Tabriz, Iran; 7 Department of Life and Health Sciences, University of Nicosia, Nicosia, Cyprus; 8 Department of Social Sciences, University of Nicosia, Nicosia, Cyprus; 9 Centre for Statistics in Medicine, NDORMS, Botnar Research Centre, University of Oxford, Oxford, UK; 10 NIHR Oxford Biomedical Research Centre, Oxford University Hospitals NHS Foundation Trust, Oxford, UK; 11 Social Determinants of Health Research Center, Shahid Beheshti University of Medical Sciences, Tehran, Iran

**Keywords:** Schizophrenia, Middle East and North Africa, Prevalence, Epidemiology, Incidence, Schizophrenia, Epidemiology

## Abstract

Schizophrenia ranks as the third-most common cause of disability among mental disorders globally. This study presents findings on the prevalence, incidence and years lived with disability (YLDs) as a result of schizophrenia in the Middle East and North Africa (MENA), stratified by age, sex and sociodemographic index (SDI). We collected publicly accessible data from the Global Burden of Disease (GBD) study 2019. This study reports the burden of schizophrenia, from 1990 to 2019, for the 21 countries that comprise MENA. In 2019, MENA exhibited an age-standardised point prevalence of 248.2, an incidence rate of 14.7 and an YLD rate of 158.7 per 100,000, which have not changed substantially between 1990 and 2019. In 2019, the age-standardised YLD rate was highest in Qatar and lowest in Afghanistan. No MENA countries demonstrated noteworthy changes in the burden of schizophrenia from 1990 to 2019. Furthermore, in 2019, the highest number of prevalent cases and the point prevalence were observed among those aged 35–39, with a higher prevalence among males in almost all age categories. Additionally, in 2019, the age-standardised YLD rates in MENA were below the worldwide average. Finally, there was a positive correlation between the burden of schizophrenia and the SDI from 1990 to 2019. The disease burden of schizophrenia has remained relatively stable over the past thirty years. Nevertheless, as the regional life-expectancy continues to increase, the burden of schizophrenia is also expected to rise. Therefore, early planning for the increase in the burden of the disease is urgently needed in the region.

## Introduction

Schizophrenia is defined as a cognitive and behavioral disorder that affects early brain development and manifests itself through several psychotic symptoms, including hallucinations, delusions, and disorganised behavior and speech^1^. The prognosis for patients with schizophrenia can vary from making a full recovery to a lifelong need for care, and patients typically have a life expectancy which is roughly twenty years less than that of the general population^1,2^. Psychiatric symptoms typically first appear during late adolescence or early adulthood, and suicidal behaviors are the most frequent cause of death early in the course of the disease^3^. Schizophrenia has also been linked to several comorbid conditions, which is partially as a result of the high prevalence of drug abuse and cigarette smoking, unhealthy lifestyles, and the potential impact of anti-psychotic medications on promoting obesity. These conditions predispose the patients to a higher rate of metabolic syndrome, diabetes, cardiovascular disorders, and respiratory diseases^4,5^.

In 2019, schizophrenia was the 42nd leading cause of disability among people of all ages and the 22nd among individuals aged 25–49 years old^6,7^. The lifetime prevalence of schizophrenia has been estimated to be just below 1%^8^. In 2019, the global age-standardised prevalence of schizophrenia was 287.4 per 100,000, and this rate was approximately the same as in 1990^6^. Also in 2019, schizophrenia accounted for 12.1% of all disability-adjusted-life-years (DALYs) attributable to mental disorders, and was surpassed only by depressive (37.4%) and anxiety (22.9%) disorders^6^. The highest incidence of schizophrenia was found in those aged 20–24, with no significant sex-based differences in the incidence rate^9^.

Several reports have been published in recent years discussing mental disorders, and more specifically the burden of schizophrenia at the regional level and across the world^6,9–13^. However, none of these articles have exclusively focused on the attributable burden of schizophrenia in the Middle East and North Africa (MENA) region. The countries located in MENA vary considerably in terms of socioeconomic profile, health system coverage and capacities, and healthcare infrastructures and provisions^14,15^. During the past three decades, the MENA region has witnessed several enhancements in health outcomes, resulting in rising life expectancies and decreased neonatal mortality^16^. Consequently, in parallel with increasing longevity, it is expected that the prevalence of chronic conditions, such as mental disorders, will continue to grow in MENA. Furthermore, as a stigmatized disease, schizophrenia is often overlooked among affected patients, especially in developing countries. Moreover, as the socioeconomic status of a country decreases the stigma of mental disorders increases, potentially leading to an underestimation of the burden of schizophrenia in lower socio-economic countries. Therefore, investigating the epidemiology of schizophrenia in the MENA region is of paramount interest^17^. Consequently, this study utilized data from the Global Burden of Disease (GBD) study 2019 to present the burden of schizophrenia in MENA from 1990 to 2019, stratified by sex, age and socio-demographic index (SDI).

## Methods

The Global Burden of Disease (GBD) study, which was established by the Institute of Health Metrics and Evaluation (IHME), measures the burden of diseases and injuries in over 200 countries and territories. Although schizophrenia is a relatively common mental problem, its burden has not been quantified across all global regions. Therefore, this study presents an assessment of the burden of schizophrenia from 1990 to 2019 for all countries in MENA. There are 21 countries in MENA, which are: Afghanistan, Algeria, Bahrain, Egypt, Iran, Iraq, Jordan, Kuwait, Lebanon, Libya, Morocco, Oman, Palestine, Qatar, Saudi Arabia, Sudan, the Syrian Arab Republic, Tunisia, Turkey, the United Arab Emirates and Yemen. A full description of the methodology utilised by IHME to model the burden of disease has been previously described^7,16,18^. The GBD 2019 estimates, which cover the period 1990–2019, are available at the following links: http://ghdx.healthdata.org/gbd-results-tool and https://vizhub.healthdata.org/gbd-compare/.

### Case definition and data sources

Schizophrenia is a serious mental disorder which is characterised by a large number of symptoms, including: delusions, hallucinations, diminished interest, flat affect, thought disorders, and emotional withdrawal. The GBD disease modelling process only included data from studies that diagnosed schizophrenia using either the Diagnostic and Statistical Manual of Mental Disorders (DSM) criteria (DSM-IV-TR: 295.10-295.30, 295.60, 295.90) or the International Classification of Diseases (ICD) criteria (ICD 10: F20). The diagnostic criteria encompass the following key elements: (1) Presence of at least two of the following symptoms, each enduring for a substantial part of a one-month period (a shorter duration if effectively treated): (i) Delusions, (ii) Hallucinations, (iii) Disorganised speech (e.g., frequent incoherence or derailment), (iv) Markedly disorganised or catatonic behavior, (v) Negative symptoms (i.e., affective flattening, alogia, or avolition); (2) Dysfunction at work and socially; (3) Persistence of the disorder’s signs and symptoms for a duration of six months or more; (4) Exclusions included substance abuse, schizoaffective and mood disorders, and/or general medical conditions, as well as any connection to pervasive developmental disorders^7^.

IHME conducted a systematic review for schizophrenia, which encompassed searching the scientific literature (i.e., PsycInfo, Embase, and PubMed), examining the grey literature, and consultation with an expert. As part of the GBD project, the electronic databases are searched biennially for mental disorders, including schizophrenia. The last systematic review for schizophrenia was carried out in GBD 2017, with the next review being due in GBD 2020. However, consulting the expert and searching the grey literature produced new data sources in GBD 2019^7^.

The inclusion criteria applied were as follows: (1) published after 1980; (2) cases were defined using DSM or ICD criteria; (3) inclusion of sufficient methodological details and sample characteristics for assessing study quality; and (4) samples that represented the general population. Specifically excluded were samples from inpatients or pharmacological treatments, case studies, veterans, or refugee cases. There were no constraints placed on the publication language. The data sources utilised to model the schizophrenia burden are accessible at this website: https://ghdx.healthdata.org/gbd-2019/data-input-sources^7^.

### Data processing and disease model

When necessary, the data extraction process involved three different age and sex splitting procedures: (1) The available estimates were divided into specific five-year age groups by sex. For example, in studies which reported the prevalence in broad age ranges separately for males and females (e.g., 15–65 year old men and women individually), and in cases where studies had smaller age groups without sex separation (e.g., prevalence among 15 to 29 year olds, then in 30 to 70 year olds, for both sexes combined), the sex ratios reported and uncertainty ranges were used to divide the age specific estimates by sex. (2) Meta-Regression with Bayesian priors, Regularisation, and Trimming (MR-BRT) was used to split the remaining data. This method involved matching sex-specific estimates for each parameter, according to location, age, and year. MR-BRT regression was then employed to model the pooled sex ratios, along with their associated uncertainty bounds. These pooled sex ratios were then utilised to split the estimates in the dataset. The prevalence ratio between males and females was 1.17 (95% uncertainty interval (UI) 0.60–1.75). 3. For prevalence estimates covering age categories spanning 25 years or more, the age pattern estimated by DisMod-MR 2.1 was used to split the data into five-year age groups. It’s important to note that the DisMod-MR model used for estimating the age pattern did not contain any previously age split data^7^.

IHME utilised DisMod MR 2.1, using the standard GBD 2019 decomposition structure, to estimate the data related to schizophrenia. At each stage of the decomposition process, IHME compared the new model with the best model from GBD 2017 and the best model from the previous stage. If substantial differences were observed between models, these variances were thoroughly explored and elucidated. In cases where it was deemed necessary, adjustments were implemented to the dataset or the model priors. When outliers were identified, they were included or excluded based upon a re-examination of their quality and methodology.

Initially, all epidemiological parameters were integrated into the modelling process. It was believed, based on the literature on schizophrenia and discussion with the expert that no cases of schizophrenia occurred before the age of 10 or after the age of 80. Furthermore, the remission rate was restricted to a maximum of 0.04, in line with the data in the dataset. In areas lacking available data, prevalence estimates were informed by location-level covariates. Only one location-level covariate, lag distributed income (LDI), was utilised to model the prevalence of schizophrenia.

### Compilation of results

The two sequelae (acute and residual) of schizophrenia, along with their corresponding disability weights (DWs), can be found in Table S1. To calculate the years lived with disability (YLDs), the prevalence estimates for each sequela were multiplied by their respective DWs. The YLDs and DALYs were the same, since there was no mortality due to schizophrenia. All estimates were standardised using the GBD standard population. 95% uncertainty intervals (UIs) were included with all estimates and were generated by producing 1000 iterations at each stage of the estimation process. The final estimates represented the mean values over the 1000 iterations, and the 95% UIs were indicated as the 25th and 975th values among the numerically ordered iterations.

Smoothing Spline models^19^ was employed to investigate the relationship the socio-demographic index (SDI) has with the burden of schizophrenia. The SDI is a composite model that contains per capita income, mean number of years attending school (aged 15 and above), and the fertility rate in women aged 25 or less. The SDI ranges from 0 to 1, representing the spectrum from the lowest to the highest development level^7^. The estimates for the point prevalence and annual incidence were obtained from the GBD website (http://ghdx.healthdata.org/gbd-results-tool) and all visual representations were created with R software (Version 3.5.2).

### Ethics approval and consent to participate

The present study was approved by Ethics Committee of Shahid Beheshti University of Medical Sciences, Tehran, Iran (IR.SBMU.RETECH.REC.1401.387).

## Results

### The Middle East and North Africa region

In 2019, there were 1.6 million (95% UI: 1.3 to 1.9) prevalent cases of schizophrenia. In addition, the age-standardised point prevalence was 248.2 (203.9 to 294.9) per 100,000, which has hardly changed since 1990 [0.5% (-1.2 to 2.0)] (Tables 1 and S2). There were 97.7 thousand (79.8 to 119.7) incident cases of schizophrenia in 2019, with an age-standardised rate of 14.7 (12.1 to 17.9) per 100,000, which did not differ from 1990 [− 1% (− 2.7 to 0.7)] (Tables 1 and S3). A total of 1.0 million (0.7 to 1.3) YLDs were attributable to schizophrenia in 2019, having an age-standardised rate of 158.7 (113.2 to 207.8) YLDs per 100,000 population. This rate also has not changed since 1990 [0.4% (− 2.2 to 3.1)] (Tables 1 and S4).

**Table 1:** Prevalent cases, incident cases and YLDAs due to schizophrenia in 2019 and percentage change in the age-standardised rates in the Middle East and North Africa region during 1990–2019.

	Prevalence (95% UI)			Incidence (95% UI)			YLDs (95% UI)		
	Counts (2019)	ASRs (2019)	Pcs in ASRs 1990–2019	Counts (2019)	ASRs (2019)	Pcs in ASRs 1990–2019	Counts (2019)	ASRs (2019)	Pcs in ASRs 1990–2019
North Africa and Middle East	1,556,694 (1,270,857, 1,863,906)	248.2 (203.9, 294.9)	0.5 (− 1.2, 2)	97,668 (79,755, 119,672)	14.7 (12.1, 17.9)	− 1 (− 2.7, 0.7)	1,000,453 (711,604, 1,312,322)	158.7 (113.2, 207.8)	0.4 (− 2.2, 3.1)
Afghanistan	59,759 (47,373, 74,507)	217.8 (176.2, 266.6)	− 2.4 (− 7.2, 2.9)	5249 (4173, 6531)	14 (11.3, 17.1)	− 1.4 (− 6.3, 3.9)	37,793 (26,352, 51,033)	135.6 (96.4, 180.8)	− 1.9 (− 10.5, 7.2)
Algeria	110,750 (87,690, 135,827)	249.1 (197.8, 304)	− 0.4 (− 5.4, 4.1)	6577 (5216, 8260)	14.8 (11.8, 18.2)	− 0.9 (− 5.8, 3.8)	71,374 (48,884, 95,646)	160 (110.9, 212.5)	− 0.6 (− 8.6, 8)
Bahrain	5391 (4285, 6662)	270.9 (216.9, 332.5)	0 (− 5.6, 5.5)	251 (199, 313)	15.5 (12.4, 19.4)	− 0.7 (− 5.9, 4.9)	3471 (2402, 4662)	173.4 (120.7, 232.3)	− 0.3 (− 8.6, 9.4)
Egypt	232,421 (185,193, 287,016)	247.6 (198.5, 302.4)	0.9 (− 4.6, 6.1)	15,460 (12,336, 19,249)	14.7 (11.8, 18.2)	− 0.6 (− 5.8, 4.4)	150,175 (103,801, 200,944)	159.1 (110.8, 211.7)	0.9 (− 8.2, 10.2)
Iran (Islamic Republic of)	247,273 (209,182, 287,365)	254.2 (216.6, 293.1)	0.8 (− 0.5, 2.1)	13,762 (11,383, 16,355)	15 (12.7, 17.7)	− 0.7 (− 2.1, 0.9)	158,115 (114,789, 202,150)	162 (118.3, 205.9)	0.9 (− 1.7, 3.2)
Iraq	95,995 (75,846, 119,639)	246.2 (196.3, 300.2)	− 1.1 (− 7, 4.2)	6988 (5535, 8802)	14.7 (11.8, 18.2)	− 1.4 (− 7.3, 3.7)	61,587 (42,426, 82,115)	156.5 (109.9, 208.9)	− 0.8 (− 9, 8)
Jordan	28,396 (22,580, 35,179)	255.1 (204.1, 312.3)	− 0.1 (− 5.6, 5.8)	1928 (1540, 2406)	15 (12.1, 18.8)	− 0.8 (− 5.8, 4.8)	18,387 (12,640, 24,958)	164 (113.9, 221.1)	− 0.2 (− 8.3, 8.8)
Kuwait	15,868 (12,431, 19,629)	273.8 (216.4, 334)	− 1 (− 6.6, 4)	834 (655, 1068)	15.5 (12.4, 19.3)	− 1.6 (− 7.6, 3.4)	10,251 (6916, 13,811)	175.6 (121, 234.3)	− 1.7 (− 10.1, 7)
Lebanon	14,020 (11,169, 17,121)	253.5 (202.5, 308.1)	0 (− 5.2, 5.8)	797 (633, 998)	14.9 (12, 18.5)	− 0.6 (− 5.7, 5.4)	8938 (6125, 11,865)	161.4 (111.1, 213.4)	− 0.2 (− 8.1, 9.1)
Libya	19,589 (15,733, 24,115)	249.9 (202, 305.3)	− 4.3 (− 9.3, 0.8)	1172 (948, 1448)	14.8 (12, 18.3)	− 3.1 (− 8.2, 2.4)	12,531 (8582, 16,833)	159.1 (109.9, 211.7)	− 5.2 (− 12.6, 3.3)
Morocco	92,573 (74,450, 112,659)	242.8 (195.3, 295.2)	0.8 (− 4.5, 5.7)	5602 (4546, 6884)	14.6 (11.8, 17.9)	− 0.6 (− 5.5, 4.5)	59,326 (41,383, 79,309)	155.3 (108.8, 206.8)	0.6 (− 7.8, 9.6)
Oman	14,884 (11,562, 18,636)	264.2 (211.5, 322.7)	− 0.1 (− 5.2, 5.6)	969 (739, 1261)	15.4 (12.3, 19.2)	− 0.7 (− 5.5, 5)	9743 (6551, 13,168)	170.4 (117.5, 226.9)	0.1 (− 8.4, 9.2)
Palestine	10,452 (8265, 12,863)	248.2 (198.5, 302.3)	0.2 (− 5.3, 5.7)	786 (625, 982)	14.8 (12, 18.2)	− 1 (− 6.2, 4.6)	6686 (4621, 9014)	157.3 (109.2, 209.7)	0.1 (− 8.6, 9.8)
Qatar	11,401 (8844, 14,469)	285 (225.4, 351.1)	0.7 (− 4.9, 6.4)	699 (528, 929)	16.2 (12.9, 20.3)	0.2 (− 5.7, 6.9)	7430 (4980, 10,159)	182.5 (125.7, 245)	0.5 (− 8.6, 9.8)
Saudi Arabia	117,603 (92,498, 146,471)	262.9 (208.4, 323.1)	− 0.8 (− 5.9, 4.5)	7085 (5562, 8923)	15.2 (12.2, 18.9)	− 1.2 (− 6.4, 4.7)	75,943 (52,479, 102,775)	167.8 (117.2, 225.2)	− 1.3 (− 9.1, 7.4)
Sudan	78,423 (61,936, 97,586)	232.7 (186.1, 284)	0.9 (− 4.7, 6.3)	6167 (4914, 7722)	14.3 (11.7, 17.7)	− 0.8 (− 5.9, 4.6)	50,721 (34,844, 68,525)	149.1 (104.2, 199.5)	0.9 (− 8.1, 10.8)
Syrian Arab Republic	34,096 (27,427, 41,729)	242.4 (194.8, 296)	− 1.3 (− 6.2, 4)	2132 (1707, 2640)	14.6 (11.8, 18)	− 1.7 (− 6.9, 3.1)	21,723 (15,179, 28,854)	154.4 (107.5, 206.2)	− 2.1 (− 9.7, 6.2)
Tunisia	33,122 (26,461, 40,293)	252.5 (201.4, 308.9)	0.4 (− 4.7, 5.7)	1757 (1416, 2180)	14.8 (12, 18.5)	− 0.8 (− 5.7, 5.2)	21,218 (14,794, 28,265)	161.9 (112.6, 216.2)	0 (− 8.7, 9.5)
Turkey	236,097 (200,869, 275,021)	248.9 (211.9, 289.5)	0.9 (− 3.8, 5.7)	13,057 (11,015, 15,495)	14.5 (12.3, 17.2)	− 0.4 (− 5.3, 4.5)	151,560 (109,660, 194,987)	159.8 (115.8, 205.5)	1.1 (− 7.2, 10.8)
United Arab Emirates	39,845 (31,205, 49,755)	275.3 (218.5, 337.2)	− 3.3 (− 9.1, 2.6)	1771 (1300, 2349)	15.7 (12.5, 19.5)	− 2.9 (− 8.6, 3.3)	25,839 (17,516, 35,640)	176.5 (123.7, 235)	− 3.6 (− 12.1, 6.1)
Yemen	57,153 (45,123, 70,636)	225.7 (180.7, 273.9)	− 2.5 (− 7.1, 2.1)	4526 (3577, 5606)	14.2 (11.4, 17.4)	− 2 (− 6.9, 3.2)	36,625 (25,366, 49,397)	143.3 (100.6, 191.3)	− 2.4 (− 10.7, 6.4)

*YLD* years lived with disability. (Generated from data available from http://ghdx.healthdata.org/gbd-results-tool).

### Country level

**Figure 1:**
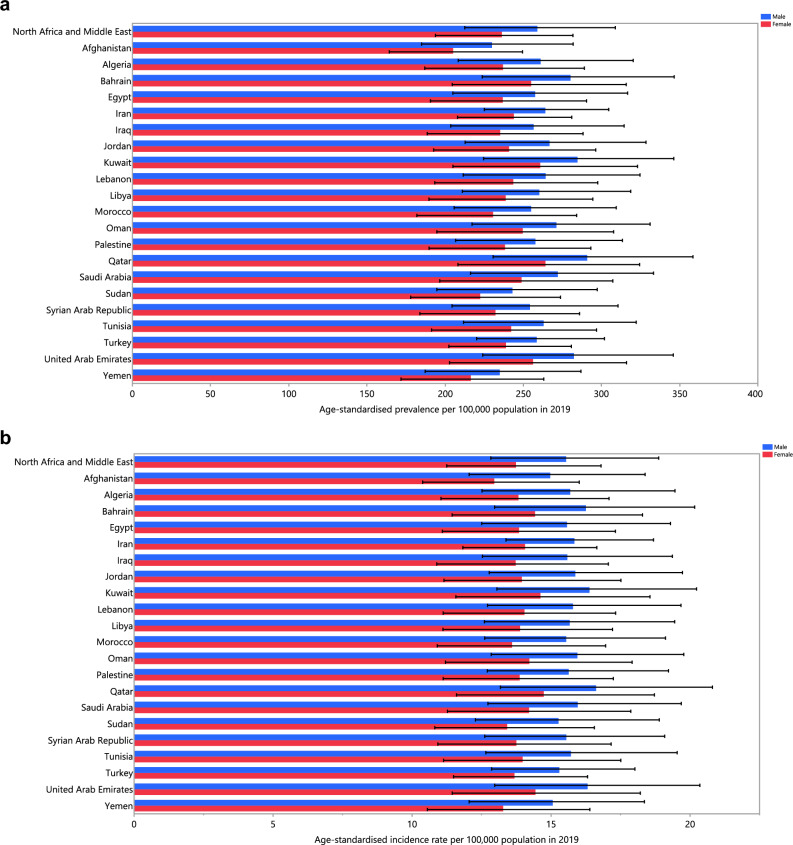

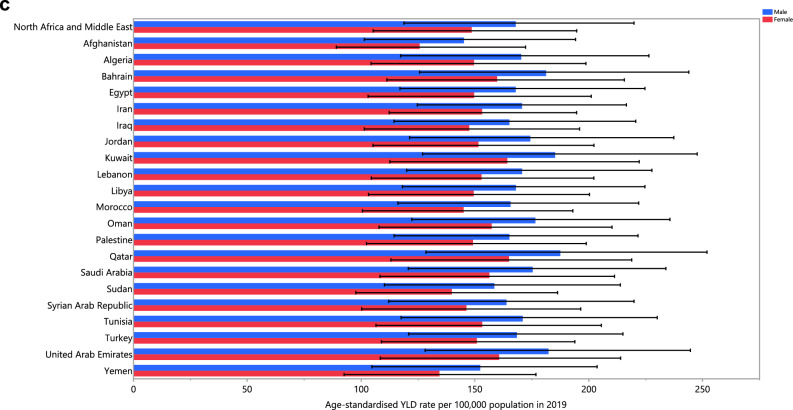
Age-standardised point prevalence (**A**), incidence rate (**B**), and YLD rate (**C**) of schizophrenia per 100,000 population in the Middle East and North Africa region in 2019, by sex and country. *YLD* years lived with disability. (Generated from data available from http://ghdx.healthdata.org/gbd-results-tool).

The age-standardised point prevalence of schizophrenia varied from 217.8 to 285.0 cases per 100,000 in the region. Qatar [285.0 (225.4 to 351.1)], the United Arab Emirates [275.3 (218.5 to 337.2)] and Kuwait [273.8 (216.4 to 334.0)] were the three highest in 2019. Conversely, Afghanistan [217.8 (176.2 to 266.6)], Yemen [225.7 (180.7 to 273.9)] and Sudan [232.7 (186.1 to 284.0)] were the three lowest (Table S2). Figure 1A presents the age-standardised point prevalence estimates of schizophrenia by country, separately for men and women, in 2019.

The age-standardised incidence rate of schizophrenia in 2019 varied from 14.0 to 16.2 cases per 100,000 in the region. Qatar [16.2 (12.9 to 20.3)], the United Arab Emirates [15.7 (12.5 to 19.5)] and Kuwait [15.5 (12.4 to 19.3)] had the highest rates, with the lowest being in Afghanistan [14.0 (11.3 to 17.1)], Yemen [14.2 (11.4 to 17.4)] and Sudan [14.3 (11.7 to 17.7)] (Table S3). Figure 1B presents the age-standardised incidence rates of schizophrenia by country, separately for males and females, in 2019.

The age-standardised YLD rate of schizophrenia in 2019 ranged from 135.6 to 182.5 cases (per 100,000) in the region. Qatar [182.5 (125.7 to 245.0)], the United Arab Emirates [176.5 (123.7 to 235.0)] and Kuwait [175.6 (121.0 to 234.3)] had the highest rates, while Afghanistan [135.6 (96.4 to 180.8)], Yemen [143.3 (100.6 to 191.3)] and Sudan [149.1 (104.2 to 199.5)] were lowest (Table S4). Figure 1C presents the age-standardised YLD rates of schizophrenia by country, separately for males and females, in 2019.

**Figure 2:**
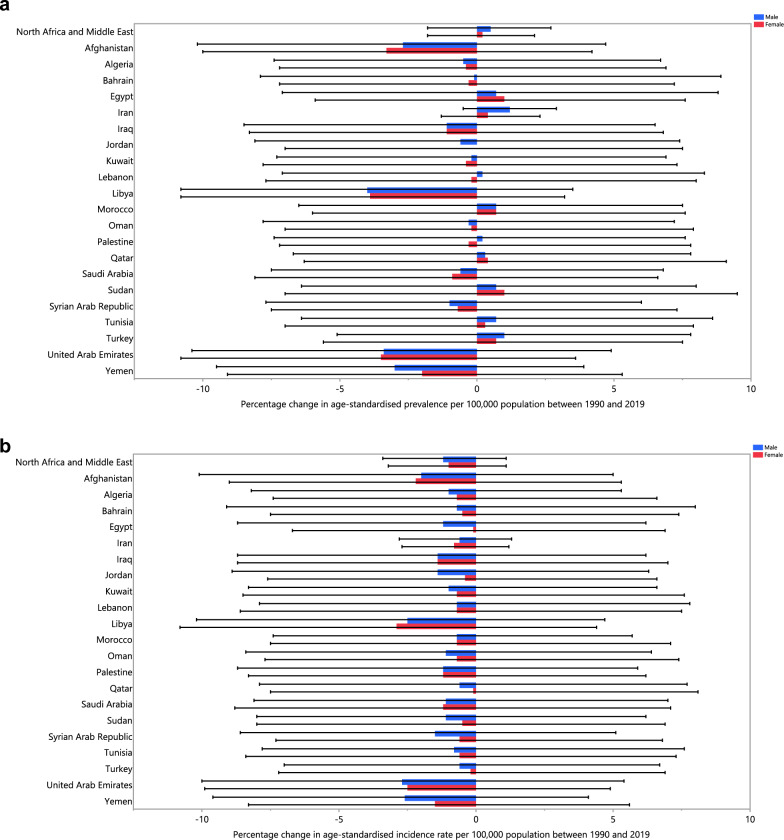

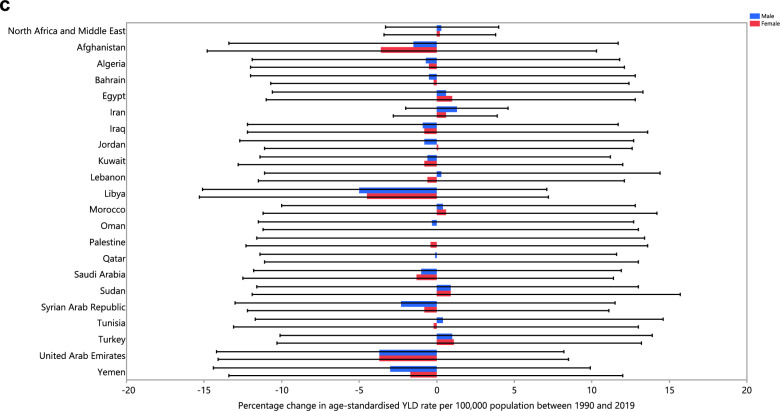
The percentage change in the age-standardised point prevalence (**A**), incidence rate (**B**), and YLD rate (**C**) of schizophrenia in the Middle East and North Africa region from 1990 to 2019, by sex and country. (Generated from data available from http://ghdx.healthdata.org/gbd-results-tool).

The age-standardised prevalence, incidence and YLD rates of schizophrenia did not change significantly in any MENA countries from 1990 to 2019 (Tables S2–S4). The changes in the age-standardised incidence, prevalence, and YLD rates for each country are depicted in Fig. 2A–C, broken down by sex, for the period 1990–2019.

### Age and sex patterns

**Figure 3:**
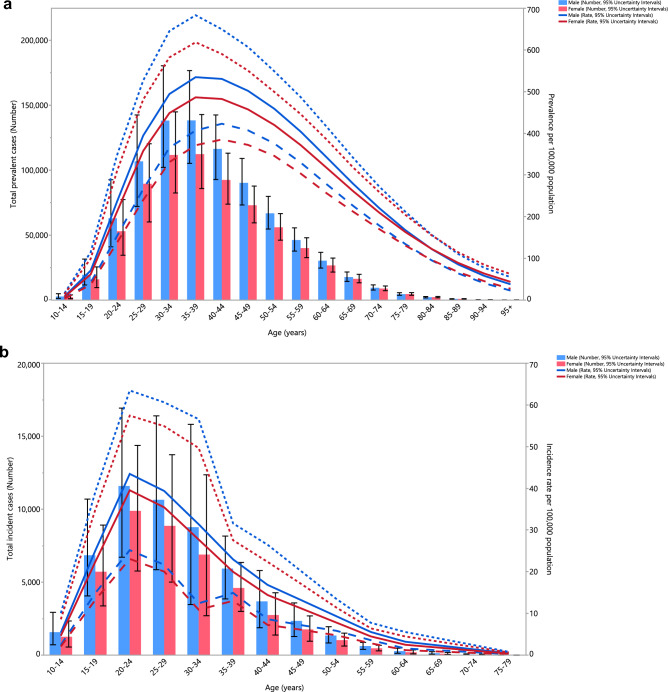

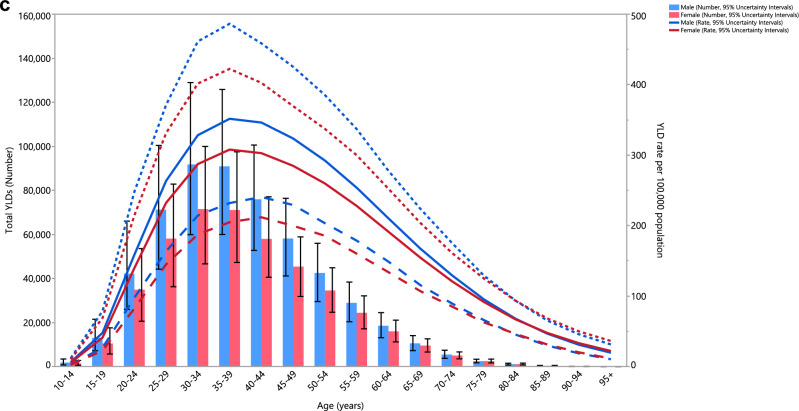
Numbers of prevalent cases and point prevalence per 100,000 population (**A**), number of incidence cases and incidence rate per 100,000 population (**B**) and the number of YLDs and YLD rate per 100,000 population (**C**) for schizophrenia in the Middle East and North Africa region, by age and sex in 2019; Dotted and dashed lines indicate 95% upper and lower uncertainty intervals, respectively. *YLD* years lived with disability. (Generated from data available from http://ghdx.healthdata.org/gbd-results-tool).

The total number of prevalent cases and the prevalence estimates in 2019 increased sharply for both sexes, starting from the 10–14 age range, reaching their highest level in those aged 35–39, before decreasing with age (Fig. 3A). Similarly, the number of incidence cases and the incidence rates began to rise from the 10–14 age range, for both sexes, were highest in the 20–24 age range and then declined with age (Fig. 3B). Furthermore, the YLD numbers rose with increasing age in both sex groups and peaked in those aged 30–34 years old, and then reduced with age. The pattern was similar for the YLD rate, but in both sexes the highest rate was seen in those aged 35–39 years old (Fig. 3C). Males had a higher prevalence, incidence and YLD cases in all age categories. Likewise, males had higher prevalence, incidence and YLD rates of schizophrenia up to 80–84 years old, while the prevalence and YLD rates were higher for females in all remaining age groups.

**Figure 4:**
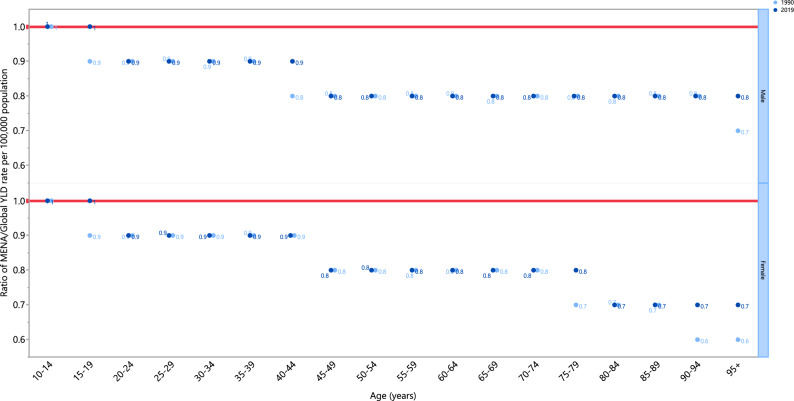
Ratio of the Middle East and North Africa region to the global schizophrenia YLD rate by age and sex, 1990 and 2019. *YLD* years lived with disability. (Generated from data available from http://ghdx.healthdata.org/gbd-results-tool).

The schizophrenia associated YLD rates in 2019 were below the global rates for both sexes over 20 years of age (ratio of MENA/global YLD rate < 1). For both sexes, people aged 10–19 years of age exhibited YLD rates that were close to the global rate (ratio of MENA/global YLD rate = 1). The YLD rate in females aged 80 and older was 0.7 times the global rate in 2019. Furthermore, in 2019 males had similar YLD ratios (ratio of MENA/global YKD rate = 1), to those in 1990, in most age groups except for 15–19, 40–44 and 95^+^ years old, which had higher ratios than in 1990. Similarly, in 2019 the YLD ratios (ratio of MENA/global YLD rate = 1) for females increased in the 15–19, 75–79 and older than 90 age-groups, compared to 1990, while all other age-groups had similar rates (Fig. 4).

### Relationship with socio-demographic index (SDI)

**Figure 5:**
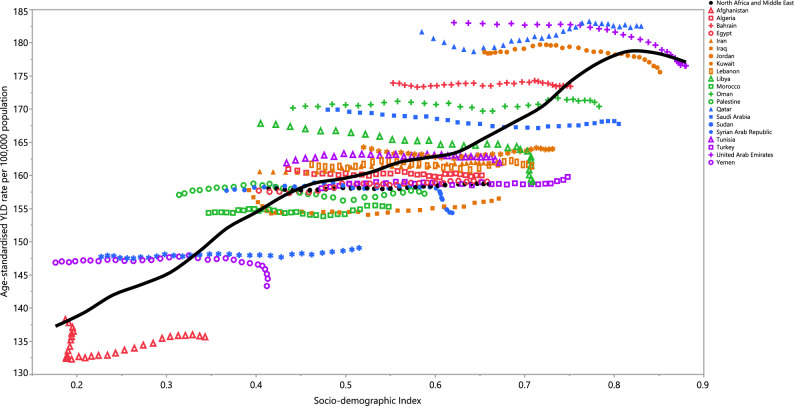
Age-standardised YLD rates of schizophrenia for 21 countries and territories, by SDI during 1990–2019; Expected values based on the Socio-demographic Index and disease rates in all locations are shown as the black line. Each point shows the observed age-standardised YLD rate for each country during 1990–2019. *YLD* years lived with disability, *SDI* Socio-demographic Index (Generated from data available from http://ghdx.healthdata.org/gbd-results-tool).

An almost linear positive association was evident between SDI and the YLD rate of schizophrenia between 1990 and 2019. In general, countries located within the region exhibited a steady rise in YLD rates, from 1990 to 2019, with increases in their SDIs. Qatar was the only country that had actual rates that were higher than those expected from 1990 to 2019, while all other countries had rates below the expected level (Fig. 5).

## Discussion

This article presents an analysis of the burden of schizophrenia in MENA, encompassing the prevalence, incidence, and YLDs, using the most recent GBD 2019 data. This study is the first to present current information on the regional and national burden of schizophrenia in the MENA region. Previous studies were either been restricted to an individual country or investigated multiple causes with limited epidemiological data.

According to the latest research on the global burden of mental diseases, schizophrenia affects far fewer patients than several other mental conditions, but the YLDs attributable to this disorder are amongst the highest of these conditions^6^. Schizophrenia presents with a wide range of clinical symptoms and signs, and also varies greatly in the severity level. Schizophrenia requires lifelong treatment, which is demanding for both the patients and their families. Furthermore, some patients may develop resistance to conventional therapies, as their condition exacerbates with more frequent relapses^20^. These patients are also at a higher risk of suicide attempts and assault, further impacting the patient, their family, and their caregivers^21,22^. Due to economic crises, rapid population growth, a shortage of healthcare staff, weak coverage, political issues, and the stigmatizing attitudes of the general population against mental illnesses, many of the healthcare systems in the MENA region are yet to reach their full potential and provide acceptable standards of care. As a result, mismanagement, misdiagnosis, or missed cases might commonly occur^23^. Thus, the true burden of schizophrenia and the disability it imposes is expected to be far higher than the estimates reported here. Drug abuse, alcoholism, and smoking are common in schizophrenic patients, which can lead to comorbidities such as malnutrition, diabetes, vascular events, blood-borne infections, and chronic obstructive pulmonary disease (COPD), causing additional disability and mortality^24^. Although these comorbidities have a global importance, the impact is even larger in economically troubled healthcare systems, which is the situation in many MENA countries. Taken together, to alleviate the burden of schizophrenia, there is an urgent need for a plan to solve the widening socioeconomic disparities and implement measures to reduce the stigma associated with schizophrenia as soon as possible.

In line with the global trend for schizophrenia, the age-standardised prevalence, incidence, and YLDs in the region did not vary significantly between 1990 and 2019^6^. In general, countries which had higher age-standardised prevalence also had higher age-standardized incidence, and YLDs (i.e., Qatar, United Arab Emirates, and Kuwait). This same pattern was also the case for the countries which showed the lowest rates (i.e. Afghanistan, Yemen, and Sudan). Moreover, schizophrenia is linked to decreased fertility in both sexes, with males experiencing a more pronounced impact^25^. This can be attributed to the behavioral and social characteristics associated with schizophrenia. It is anticipated that decreased fertility will increase due to the ongoing delayed marriage patterns, even though the age of onset for schizophrenia will remain unchanged^26^. Natural selection is expected to reduce the population frequencies of genes associated with reduced fertility. Nonetheless, the prevalence of schizophrenia continues to be high, not only in the MENA region but also globally, with the frequency of the disease showing no significant change in recent decades^27^. This is commonly known as a "Darwinian paradox"^26^. Multiple hypotheses have been proposed to explain how schizophrenia evades the influence of natural selection, but the exact mechanism remains an enigma^28–30^. A plausible explanation for the unchanged prevalence of schizophrenia, despite its association with decreased fertility, is that the genetic factors contributing to schizophrenia may also confer advantages related to the development of essential human characteristics, including language, complex cognitive skills, and other favorable brain functions^31^. This hypothesis is substantiated by the presence of enhanced recent evolutionary markers near the loci linked to schizophrenia^31,32^. However, the evolutionary puzzle of schizophrenia remains complex and requires further research to be fully understood.

As illustrated in Fig. 2A–C, the highest incidence of schizophrenia was observed in the 15 to 39 age group, and the disease’s prevalence peaked among those aged 20 to 54 years old, after which it gradually decreased with increasing age. The peak incidence starts earlier in life (20 to 24 age group) and the prevalence peaks in the 35 to 39 age group, and then reduces with age. This pattern was also seen for the YLD rates. The presented data emphasises the need for screening and intervention before the peak ages in the incidence, and also underlines the increased need for social, mental, and healthcare support during the peaks in the prevalence and YLDs. As the disease gets more chronic, and particularly when accompanied by more frequent relapses (either due to the nature of the disease or by mismanagement), more YLDs are observed and thus more access to medical care and social support is required to prevent treatment resistant conditions and worse outcomes, such as suicide, overdose, or domestic violence^33^. In almost all age groups, men showed higher prevalence, incidence and YLD values and rates, but these differences were not statistically significant. The changes in incidence, prevalence, and YLDs observed in both sexes generally show a decrease from 1990 to 2019 in most countries. Interestingly, the percentage changes in the incidence were negative in all MENA countries. Nevertheless, none of the changes were statistically significant, and thus should be carefully interpreted with regards to future planning and policy making.

The MENA YLD rates were below those found globally for all age groups, with the exception of those aged 10 to 19 year olds. This can be explained through the vast medical and non-medical problems faced by most countries in MENA. The burden of communicable diseases are substantially higher in MENA, than globally, and thus chronic conditions such as mental disorders might not receive the appropriate priority level for their management and treatment^34^. Furthermore, the burden of schizophrenia remained unchanged from 1990 to 2019 in most age groups, except for the elderly ages, which have increased.

As displayed in Fig. 4, SDI has a positive linear relationship with the age-standardised YLD rate in MENA. These results should be carefully interpreted as there are major gaps between the countries showing the lowest values and those with the highest. Countries such as Afghanistan, Yemen, and Sudan were embroiled in prolonged conflicts during much of the measurement period, and their healthcare systems have been severely affected by their unbalanced economies and political problems^35,36^. Consequently, the low burden of schizophrenia in these countries is likely to be highly biased and artificially underestimated. In contrast, economically stable and high-income countries in this region have shown a higher burden of schizophrenia, which can be attributed to their more efficient healthcare systems and screening strategies. An alternative explanation for this finding might be that the high level of urbanisation and high density housing in the high income countries is related to the higher incidence of schizophrenia, due to elevated levels of stress and pollution in these areas^37,38^. While GBD continues to improve on the data and methodologies for estimating the burden of mental disorders, including schizophrenia, several challenges need acknowledging. Firstly, there were a large number of locations without high-quality raw data. Secondly, quantifying and eliminating all variation caused by measurement error in our prevalence estimates is a challenging task. Although IHME has refined the methodology to address known sources of bias (e.g., case definitions or survey methods), there are still very few data points available to inform such adjustments. Additionally, there is a paucity of research on the risk factors of mental disorders which can be used as predictive covariates in our epidemiological models^39^.

## Conclusion

The present article highlights the importance of cautiously interpreting the currently available epidemiological information on the burden of schizophrenia in MENA, since the gathered data are prone to several biases. Thus, presumably the low burden of this condition might increase substantially in the future, as the healthcare systems start to screen and identify more patients. The most important aspect in preventing any future rise in the burden of schizophrenia lies in the efficient screening and prompt identification of patients, and then effectively treating these patients using a holistic approach. By reducing the prevalence of this mental condition, the burden of its related comorbidities and problems will also be addressed, significantly contributing to the overall health of the communities and the countries. Finally, it is important not to underestimate the significance of stigma directed towards people with psychiatric disorders. Initiatives aimed at increasing awareness about schizophrenia among patients, their families and their social networks can contribute significantly to reducing the disability associated with the disease.

### Supplementary Information

Supplementary Tables.

## Data Availability

The data used for these analyses are all publicly available at http://ghdx.healthdata.org/gbd-results-tool.
