# Supplementary material for: The burden of schizophrenia in the Middle East and North Africa region, 1990–2019
Source: Sci Rep. 2024 Apr 27;14:9720. doi: 10.1038/s41598-024-59905-8 (PMC11055947; doi:10.1038/s41598-024-59905-8)
Supplement: Supplementary file 1 — Supplementary Tables. [file 41598_2024_59905_MOESM1_ESM.docx]

| **Table S1: The severity levels, lay descriptions and disability weights (DWs) of schizophrenia in the Global Burden of Disease Study 2019** | | |
| --- | --- | --- |
| **Severity level** | **Lay description** | **Disability weight**  **(95% CI)** |
| Acute state | Hears and sees things that are not real and is afraid, confused, and sometimes violent. The person has great difficulty with communication and daily activities, and sometimes wants to harm or kill himself (or herself). | 0.778  (0.606 – 0.9) |
| Residual state | Hears and sees things that are not real and has trouble communicating. The person can be forgetful, has difficulty with daily activities, and thinks about hurting himself (or herself). | 0.588  (0.411 – 0.754) |
| CI: Confidence interval | |  |

| **Table S2: Prevalence of schizophrenia in 1990 and 2019 and the percentage change in the age-standardised rates (ASRs) per 100,000 in the North Africa and the Middle East region**  **(Generated from data available from http://ghdx.healthdata.org/gbd-results-tool)** | | | | | |
| --- | --- | --- | --- | --- | --- |
|  | **1990** | | **2019** | | **Percentage change in ASRs per 100,000** |
|  | **No (95% UI)** | **ASRs per 100,000 (95% UI)** | **No (95% UI)** | **ASRs per 100,000 (95% UI)** |  |
| **North Africa and Middle East** | **661097 (538401 , 795971)** | **247 (204.3 , 292.8)** | **1556694 (1270857 , 1863906)** | **248.2 (203.9 , 294.9)** | **0.5 (-1.2 , 2)** |
| **Afghanistan** | **17880 (14368 , 21845)** | **223.2 (177.8 , 273.1)** | **59759 (47373 , 74507)** | **217.8 (176.2 , 266.6)** | **-2.4 (-7.2 , 2.9)** |
| **Algeria** | **47041 (36924 , 59040)** | **250.2 (198.9 , 306.5)** | **110750 (87690 , 135827)** | **249.1 (197.8 , 304)** | **-0.4 (-5.4 , 4.1)** |
| **Bahrain** | **1399 (1079 , 1773)** | **271 (216.4 , 331.4)** | **5391 (4285 , 6662)** | **270.9 (216.9 , 332.5)** | **0 (-5.6 , 5.5)** |
| **Egypt** | **111414 (87874 , 139075)** | **245.4 (196.4 , 300.8)** | **232421 (185193 , 287016)** | **247.6 (198.5 , 302.4)** | **0.9 (-4.6 , 6.1)** |
| **Iran (Islamic Republic of)** | **107470 (90645 , 125960)** | **252.2 (214.7 , 291.2)** | **247273 (209182 , 287365)** | **254.2 (216.6 , 293.1)** | **0.8 (-0.5 , 2.1)** |
| **Iraq** | **30697 (24190 , 38297)** | **248.8 (200.1 , 306.2)** | **95995 (75846 , 119639)** | **246.2 (196.3 , 300.2)** | **-1.1 (-7 , 4.2)** |
| **Jordan** | **6748 (5309 , 8383)** | **255.3 (205.7 , 309.9)** | **28396 (22580 , 35179)** | **255.1 (204.1 , 312.3)** | **-0.1 (-5.6 , 5.8)** |
| **Kuwait** | **4913 (3812 , 6209)** | **276.6 (220.4 , 339.9)** | **15868 (12431 , 19629)** | **273.8 (216.4 , 334)** | **-1 (-6.6 , 4)** |
| **Lebanon** | **6989 (5564 , 8626)** | **253.6 (201.5 , 310.1)** | **14020 (11169 , 17121)** | **253.5 (202.5 , 308.1)** | **0 (-5.2 , 5.8)** |
| **Libya** | **7916 (6257 , 9835)** | **261.1 (210.2 , 319.4)** | **19589 (15733 , 24115)** | **249.9 (202 , 305.3)** | **-4.3 (-9.3 , 0.8)** |
| **Morocco** | **49911 (39546 , 62056)** | **241 (193.2 , 293.9)** | **92573 (74450 , 112659)** | **242.8 (195.3 , 295.2)** | **0.8 (-4.5 , 5.7)** |
| **Oman** | **4220 (3272 , 5291)** | **264.4 (210.9 , 323)** | **14884 (11562 , 18636)** | **264.2 (211.5 , 322.7)** | **-0.1 (-5.2 , 5.6)** |
| **Palestine** | **3359 (2651 , 4181)** | **247.7 (197.2 , 302)** | **10452 (8265 , 12863)** | **248.2 (198.5 , 302.3)** | **0.2 (-5.3 , 5.7)** |
| **Qatar** | **1438 (1099 , 1832)** | **282.9 (225.3 , 348.8)** | **11401 (8844 , 14469)** | **285 (225.4 , 351.1)** | **0.7 (-4.9 , 6.4)** |
| **Saudi Arabia** | **33607 (26421 , 42131)** | **264.9 (213.8 , 324.9)** | **117603 (92498 , 146471)** | **262.9 (208.4 , 323.1)** | **-0.8 (-5.9 , 4.5)** |
| **Sudan** | **33601 (26747 , 41609)** | **230.7 (186.1 , 280.8)** | **78423 (61936 , 97586)** | **232.7 (186.1 , 284)** | **0.9 (-4.7 , 6.3)** |
| **Syrian Arab Republic** | **21365 (16873 , 26844)** | **245.6 (196.4 , 301.3)** | **34096 (27427 , 41729)** | **242.4 (194.8 , 296)** | **-1.3 (-6.2 , 4)** |
| **Tunisia** | **17568 (13892 , 21807)** | **251.4 (200.6 , 305.5)** | **33122 (26461 , 40293)** | **252.5 (201.4 , 308.9)** | **0.4 (-4.7 , 5.7)** |
| **Turkey** | **127319 (107889 , 149864)** | **246.8 (210.8 , 288.6)** | **236097 (200869 , 275021)** | **248.9 (211.9 , 289.5)** | **0.9 (-3.8 , 5.7)** |
| **United Arab Emirates** | **5711 (4380 , 7271)** | **284.8 (225.9 , 351.2)** | **39845 (31205 , 49755)** | **275.3 (218.5 , 337.2)** | **-3.3 (-9.1 , 2.6)** |
| **Yemen** | **20087 (15976 , 24984)** | **231.5 (185.9 , 283.4)** | **57153 (45123 , 70636)** | **225.7 (180.7 , 273.9)** | **-2.5 (-7.1 , 2.1)** |

| **Table S3: Incidence of schizophrenia in 1990 and 2019 and the percentage change in the age-standardised rates (ASRs) per 100,000 in the North Africa and the Middle East region**  **(Generated from data available from http://ghdx.healthdata.org/gbd-results-tool)** | | | | | |
| --- | --- | --- | --- | --- | --- |
|  | **1990** | | **2019** | | **Percentage change in ASRs per 100,000** |
|  | **No (95% UI)** | **ASRs per 100,000 (95% UI)** | **No (95% UI)** | **ASRs per 100,000 (95% UI)** |  |
| **North Africa and Middle East** | **50545 (41106 , 61931)** | **14.8 (12.3 , 18)** | **97668 (79755 , 119672)** | **14.7 (12.1 , 17.9)** | **-1 (-2.7 , 0.7)** |
| **Afghanistan** | **1399 (1096 , 1742)** | **14.2 (11.5 , 17.5)** | **5249 (4173 , 6531)** | **14 (11.3 , 17.1)** | **-1.4 (-6.3 , 3.9)** |
| **Algeria** | **3764 (2963 , 4757)** | **14.9 (12 , 18.5)** | **6577 (5216 , 8260)** | **14.8 (11.8 , 18.2)** | **-0.9 (-5.8 , 3.8)** |
| **Bahrain** | **98 (76 , 128)** | **15.6 (12.5 , 19.4)** | **251 (199 , 313)** | **15.5 (12.4 , 19.4)** | **-0.7 (-5.9 , 4.9)** |
| **Egypt** | **8228 (6575 , 10364)** | **14.8 (11.9 , 18.5)** | **15460 (12336 , 19249)** | **14.7 (11.8 , 18.2)** | **-0.6 (-5.8 , 4.4)** |
| **Iran (Islamic Republic of)** | **8517 (7074 , 10161)** | **15.1 (12.7 , 17.8)** | **13762 (11383 , 16355)** | **15 (12.7 , 17.7)** | **-0.7 (-2.1 , 0.9)** |
| **Iraq** | **2432 (1923 , 3069)** | **14.9 (12 , 18.4)** | **6988 (5535 , 8802)** | **14.7 (11.8 , 18.2)** | **-1.4 (-7.3 , 3.7)** |
| **Jordan** | **583 (453 , 738)** | **15.1 (12.1 , 18.7)** | **1928 (1540 , 2406)** | **15 (12.1 , 18.8)** | **-0.8 (-5.8 , 4.8)** |
| **Kuwait** | **343 (264 , 440)** | **15.8 (12.7 , 19.6)** | **834 (655 , 1068)** | **15.5 (12.4 , 19.3)** | **-1.6 (-7.6 , 3.4)** |
| **Lebanon** | **469 (374 , 582)** | **15 (12.1 , 18.6)** | **797 (633 , 998)** | **14.9 (12 , 18.5)** | **-0.6 (-5.7 , 5.4)** |
| **Libya** | **625 (497 , 785)** | **15.3 (12.3 , 18.9)** | **1172 (948 , 1448)** | **14.8 (12 , 18.3)** | **-3.1 (-8.2 , 2.4)** |
| **Morocco** | **3814 (3020 , 4752)** | **14.7 (11.8 , 18.1)** | **5602 (4546 , 6884)** | **14.6 (11.8 , 17.9)** | **-0.6 (-5.5 , 4.5)** |
| **Oman** | **307 (239 , 388)** | **15.5 (12.4 , 19.1)** | **969 (739 , 1261)** | **15.4 (12.3 , 19.2)** | **-0.7 (-5.5 , 5)** |
| **Palestine** | **284 (222 , 358)** | **14.9 (12 , 18.5)** | **786 (625 , 982)** | **14.8 (12 , 18.2)** | **-1 (-6.2 , 4.6)** |
| **Qatar** | **93 (71 , 121)** | **16.1 (12.9 , 20.1)** | **699 (528 , 929)** | **16.2 (12.9 , 20.3)** | **0.2 (-5.7 , 6.9)** |
| **Saudi Arabia** | **2583 (2038 , 3282)** | **15.4 (12.3 , 19.4)** | **7085 (5562 , 8923)** | **15.2 (12.2 , 18.9)** | **-1.2 (-6.4 , 4.7)** |
| **Sudan** | **2732 (2168 , 3411)** | **14.4 (11.6 , 17.8)** | **6167 (4914 , 7722)** | **14.3 (11.7 , 17.7)** | **-0.8 (-5.9 , 4.6)** |
| **Syrian Arab Republic** | **1779 (1401 , 2243)** | **14.8 (12 , 18.4)** | **2132 (1707 , 2640)** | **14.6 (11.8 , 18)** | **-1.7 (-6.9 , 3.1)** |
| **Tunisia** | **1301 (1020 , 1628)** | **15 (12 , 18.4)** | **1757 (1416 , 2180)** | **14.8 (12 , 18.5)** | **-0.8 (-5.7 , 5.2)** |
| **Turkey** | **9132 (7637 , 10778)** | **14.6 (12.2 , 17.1)** | **13057 (11015 , 15495)** | **14.5 (12.3 , 17.2)** | **-0.4 (-5.3 , 4.5)** |
| **United Arab Emirates** | **378 (288 , 492)** | **16.1 (12.8 , 20.2)** | **1771 (1300 , 2349)** | **15.7 (12.5 , 19.5)** | **-2.9 (-8.6 , 3.3)** |
| **Yemen** | **1650 (1306 , 2073)** | **14.5 (11.7 , 17.7)** | **4526 (3577 , 5606)** | **14.2 (11.4 , 17.4)** | **-2 (-6.9 , 3.2)** |

| **Table S4: YLDs due to schizophrenia in 1990 and 2019 for both sexes and percentage change in the age-standardised rates (ASRs) per 100,000 in the North Africa and the Middle East region**  **(Generated from data available from http://ghdx.healthdata.org/gbd-results-tool)** | | | | | |
| --- | --- | --- | --- | --- | --- |
|  | **1990** | | **2019** | | **Percentage change in ASRs per 100,000** |
|  | **No (95% UI)** | **ASRs per 100,000 (95% UI)** | **No (95% UI)** | **ASRs per 100,000 (95% UI)** |  |
| **North Africa and Middle East** | **426301 (302521 , 562470)** | **158.1 (113.8 , 206.5)** | **1000453 (711604 , 1312322)** | **158.7 (113.2 , 207.8)** | **0.4 (-2.2 , 3.1)** |
| **Afghanistan** | **11113 (7803 , 14861)** | **138.2 (97.1 , 184.6)** | **37793 (26352 , 51033)** | **135.6 (96.4 , 180.8)** | **-1.9 (-10.5 , 7.2)** |
| **Algeria** | **30512 (21033 , 41491)** | **161 (112.3 , 215.1)** | **71374 (48884 , 95646)** | **160 (110.9 , 212.5)** | **-0.6 (-8.6 , 8)** |
| **Bahrain** | **911 (620 , 1235)** | **173.9 (120.4 , 231.3)** | **3471 (2402 , 4662)** | **173.4 (120.7 , 232.3)** | **-0.3 (-8.6 , 9.4)** |
| **Egypt** | **72105 (50084 , 96712)** | **157.7 (110.1 , 210.9)** | **150175 (103801 , 200944)** | **159.1 (110.8 , 211.7)** | **0.9 (-8.2 , 10.2)** |
| **Iran (Islamic Republic of)** | **69046 (50674 , 88190)** | **160.6 (118.1 , 203.4)** | **158115 (114789 , 202150)** | **162 (118.3 , 205.9)** | **0.9 (-1.7 , 3.2)** |
| **Iraq** | **19657 (13492 , 26583)** | **157.8 (109.6 , 210.7)** | **61587 (42426 , 82115)** | **156.5 (109.9 , 208.9)** | **-0.8 (-9 , 8)** |
| **Jordan** | **4393 (3041 , 5893)** | **164.3 (114.5 , 219.8)** | **18387 (12640 , 24958)** | **164 (113.9 , 221.1)** | **-0.2 (-8.3 , 8.8)** |
| **Kuwait** | **3211 (2163 , 4353)** | **178.6 (123.3 , 239.5)** | **10251 (6916 , 13811)** | **175.6 (121 , 234.3)** | **-1.7 (-10.1 , 7)** |
| **Lebanon** | **4471 (3123 , 5940)** | **161.7 (113.6 , 213.8)** | **8938 (6125 , 11865)** | **161.4 (111.1 , 213.4)** | **-0.2 (-8.1 , 9.1)** |
| **Libya** | **5136 (3455 , 6897)** | **167.8 (114.1 , 224.6)** | **12531 (8582 , 16833)** | **159.1 (109.9 , 211.7)** | **-5.2 (-12.6 , 3.3)** |
| **Morocco** | **32204 (22537 , 42889)** | **154.4 (107.2 , 204.2)** | **59326 (41383 , 79309)** | **155.3 (108.8 , 206.8)** | **0.6 (-7.8 , 9.6)** |
| **Oman** | **2753 (1874 , 3722)** | **170.2 (117.8 , 226.3)** | **9743 (6551 , 13168)** | **170.4 (117.5 , 226.9)** | **0.1 (-8.4 , 9.2)** |
| **Palestine** | **2149 (1487 , 2898)** | **157.1 (110.2 , 210.3)** | **6686 (4621 , 9014)** | **157.3 (109.2 , 209.7)** | **0.1 (-8.6 , 9.8)** |
| **Qatar** | **939 (632 , 1297)** | **181.7 (126.2 , 240.7)** | **7430 (4980 , 10159)** | **182.5 (125.7 , 245)** | **0.5 (-8.6 , 9.8)** |
| **Saudi Arabia** | **21838 (15100 , 29631)** | **169.9 (117.8 , 228.4)** | **75943 (52479 , 102775)** | **167.8 (117.2 , 225.2)** | **-1.3 (-9.1 , 7.4)** |
| **Sudan** | **21689 (15280 , 29328)** | **147.7 (104.2 , 197.1)** | **50721 (34844 , 68525)** | **149.1 (104.2 , 199.5)** | **0.9 (-8.1 , 10.8)** |
| **Syrian Arab Republic** | **13848 (9539 , 18610)** | **157.7 (111.7 , 209.5)** | **21723 (15179 , 28854)** | **154.4 (107.5 , 206.2)** | **-2.1 (-9.7 , 6.2)** |
| **Tunisia** | **11383 (7910 , 15250)** | **161.9 (113.8 , 214.7)** | **21218 (14794 , 28265)** | **161.9 (112.6 , 216.2)** | **0 (-8.7 , 9.5)** |
| **Turkey** | **82068 (59833 , 104256)** | **158.1 (115 , 200.8)** | **151560 (109660 , 194987)** | **159.8 (115.8 , 205.5)** | **1.1 (-7.2 , 10.8)** |
| **United Arab Emirates** | **3734 (2490 , 5136)** | **183 (127 , 245.2)** | **25839 (17516 , 35640)** | **176.5 (123.7 , 235)** | **-3.6 (-12.1 , 6.1)** |
| **Yemen** | **12853 (8874 , 17406)** | **146.9 (102.7 , 196.6)** | **36625 (25366 , 49397)** | **143.3 (100.6 , 191.3)** | **-2.4 (-10.7 , 6.4)** |

.
